# Annual Meeting of the Virginia State Dental Association

**Published:** 1878-01

**Authors:** J. B. Hodgkin


### ARTICLE II.

### *Annual Meeting of the Virginia State Dental Association.*

[Reported for this Journal by Prof. J. B. Hodgkin.]

The Eighth Annual Meeting of the Virginia State Dental Association was convened in Richmond, Va., Dec. 11th, the President, Dr. W. W. H. Thackston, in the chair.

Quite a number of dentists from various parts of the State were present. The first few hours of the session were consumed in routine business, and the Association adjourned until the next day at 10 A. M.

SECOND DAY—MORNING.

The Association was called to order by the President, and after the usual routine business, the names of Drs. Wm. Farmer, of Wytheville, N. M. Burkholder, of Harrisonburg, D. N. Rust, of Alexandria, J. O. Hodgkin, of Warrenton, and R. Y. Henly, of King and Queen County, were reported for membership and these gentlemen were elected.

Dr. Keesee read a letter from Dr. H. M. Grant, of Va., regretting his inability to attend, and assuring the Association of his cordial sympathy with its aims.

The President of the Association, Dr. Thackston, then read his Annual Address, which will be published in the next number of this Journal.

At the conclusion of the address, which was listened to with deep attention and loudly applauded, Dr. Steele, of Richmond, said that for one he was thankful to the President for making the able and eloquent address on this subject. It chimed in with his own feelings and thoughts. Why could not the dental profession be protected in Virginia, as well as in other States from the tramps and charlatans which infest it? He trusted that a way would be opened by which a remedy would be found for the evils so graphically described in the address, and he hoped that some means would soon be found of protection against the ignorant pretenders who had been run out of their own

State by the wholesome legislative enactments which there protected the dentists. He moved the acceptance of the report, with the thanks of the Association to Dr. Thackston, and that a committee be appointed to inquire into the expediency of memorializing the State Legislature for the protection of the Dentists of this State. The subject was, after further discussion, referred to a committee consisting of Drs. Steele, Henkle, Thompson and Moore.

The Executive Committee reported through Dr. L. M. Cowardin, that they had made arrangements for clinics, in case any member should desire to operate, and that they had also made suitable arrangements for the reception and accommodation of visitors.

On the subject of Operative Dentistry, Dr. Thompson stated that had not had opportunity of conferring with the other members of that committee, and so had no formal concerted report. He read a brief paper he had hastily prepared, in lieu of anything more formal. [Will be published in Feb. number of Journal.]

The subject of Operative Dentistry being now open for discussion:

Dr. Johnston, of Staunton, stated that as to sensitive dentine, he had long ago ceased to use any of the many things recommended as obtunders of sensibility. He had found that moral courage and sharp instruments did more than anything else to overcome these difficulties. The confidence of our patients was a great help also. If they had faith in us and our methods of work, it greatly enabled them to bear pain.

Dr. Keesee spoke of the subject of Nervous Exhaustion, which was one of the topics treated of in the paper of Dr. Thompson. He was told by a friend of his that he had found benefit by insulating himself from his patients by the use of a silk garment.

Prof. Hodgkin thought there was a mistake usually made as to the nature of the trouble spoken of. If members would agree to call it "brain worry," it would be more properly defined. The fact that some patients exhausted us more than others, arose he thought, from the fact that they gave us more trouble, more anxiety and uneasiness; we found them difficult to operate for, nervous and suffering themselves, and we had trouble in all the steps of our work. The condition of the patient presented obstacles in the way of the easy performance of the, under any circumstances, difficult operation, and we had the condition known as "*brain worry*" brought on. *Brain work* was not nearly so exhaustive, and more easily recovered from ; *brain worry* is debilitating.

Dr. Johnston replied, that this was doubtless a solution of the difficulty, at least in many cases.

In further discussing the paper, he spoke of the trouble he had formerly experienced in filling nerve canals, and he had lately been indebted to Dr. Moore, of Richmond, for a method of filling roots of teeth, which was, in his hands, giving a greater amount of satisfaction than any method he had ever seen pursued, very greatly lessening the labor and uncertainty of root filling. The method was to make an instrument of block tin, about the size and length of an ordinary excavator, and shape the end of this to the canal approximately. This part fitting the root, was made rough by a coarse file, and pushed up into the canal. The friction of the walls of the pulp canal against the tin made it somewhat smooth at the points at which it fitted most tightly, and on withdrawing it, this fact was taken advantage of to lessen its size, and by a few trials the tapering tin broach could be accurately fitted to the entire length of the root. It was now marked the proper length and cut off, and a very little of the point or upper extremity also cut off, and on being placed in position could be driven into the canal, with a slight stroke of the mallet. He never enlarged a nerve canal, but by this method of adjustment, found the work of filling roots so simplified as to reduce the trouble to a minimum.

Dr. Henkle said that he had tried this method of filling roots, and corroborated all that Dr. Johnston had said as to ts efficiency and simplicity. It was, of all the plans he had tried—and he had tried very many—the most practicable.

Dr. Keesee wished to know, if this was a better method than to fill with Hill's stopping?

Dr. Moore replied, that he had practised this method of filling roots for seven years, and had experimented largely on teeth out of the mouth, with gold and tin and other methods. He had found on cutting down to the filling, that the root was more perfectly filled this way than any other he had seen. This method had the great additional advantage that the filling could be easily removed, in case it was desirable or necessary to do so. He adverted to the nervous exhaustion experienced in working for some patients, but did not think the explanation of Prof. Hodgkin a sufficient one. He had found himself as thoroughly used up by quiet patients, with simple work, as by the more restless ones, with difficult cavities. Many patients gave him no trouble, while others with no worse teeth to fill, seemed to take away all his vitality. He was willing to admit, however, that brain worry had a great deal to do with the breaking down of the constitutions of a great many of the members of our profession.

Dr. Cowardin discussed the subject of Sensitive Dentine, to which allusion was made in Dr. Thompson's paper. In his own case while at college, his front teeth which required filling, were so exquisitely sensitive as almost to throw him into convulsions. All remedies having failed, recourse was had to the rhigoline spray, which was used with great satisfaction indeed, as an obtunder. Since that time he had frequently used the spray apparatus in his practice, for the like purpose with similarly satisfactory results. He said in answer to a question, that his teeth were sensitive for a long time to changes of temperature, as might be expected, but that this gradually wore away, and now they were very comfortable. The lowering of the temperature was not pushed to the point of danger, but only sufficiently to render bearable an otherwise unendurable pain.

Dr. Thompson corroborated Dr. Cowardin in his remarks on the beneficial use of the spray, so far as sensitive dentine was concerned.

He added that he had laid aside its use in extracting, finding it inefficient. He was much interested in some experiments on what was termed Atmospheric Anæsthesia, or partial anæsthesia produced by rapid and forcible inhalation of air, and would like to know if it had been tried by the profession, and with what results. For himself he had secured results this way, which were certainly very extraordinary, and had performed operations by the aid of this method, which he could not he thought, have performed without some help of this sort.

Dr. Scribner said, that he apprehended the true solution of the effect here produced was in the distraction of the attention of the patient. Whatever would do this would help greatly. Where attention was concentrated on any one point, to that point there was nervous flow.

Dr. Henkle said that Dr. Bonwill was the first to suggest this method. He himself had tried it with favorable results. The question of diversion of the attention however had weight. The simple use on the part of the patient, of a saliva pump, often seemed to answer this purpose.

Dr. Sprenkel had found that perfect dryness helped greatly. By the rubber dam and a current of hot air, he succeeded in managing most of his cases.

Dr. Moore thought that the health of the dentist was often injured, by a fact that we all recognised—all our work was troublesome, and to honestly perform it, it involved an amount of care and anxiety which was trying on the constitution. He had tried carbolic acid and glycerine as an obtunder with favorable results.

[TO BE CONTINUED.]

